# Resistance mechanism of human immunodeficiency virus type-1 protease to inhibitors: A molecular dynamic approach

**Published:** 2014-12

**Authors:** Mohammad Reza Dayer, Mohammad Saaid Dayer

**Affiliations:** 1 Department of Biology, Faculty of Science, Shahid Chamran University of Ahvaz, Iran; 2 Department of Parasitology and Medical Entomology, Tarbiat Modares University, Tehran, Iran

**Keywords:** HIV-1 Protease, Inhibitors, AIDS Treatment, Drug Resistance

## Abstract

Human immunodeficiency virus type 1 (HIV-1) protease inhibitors comprise an important class of drugs used in HIV treatments. However, mutations of protease genes accelerated by low fidelity of reverse transcriptase yield drug resistant mutants of reduced affinities for the inhibitors. This problem is considered to be a serious barrier against HIV treatment for the foreseeable future. In this study, molecular dynamic simulation method was used to examine the combinational and additive effects of all known mutations involved in drug resistance against FDA approved inhibitors. Results showed that drug resistant mutations are not randomly distributed along the protease sequence; instead, they are localized on flexible or hot points of the protein chain. Substitution of more hydrophobic residues in flexible points of protease chains tends to increase the folding, lower the flexibility and decrease the active site area of the protease. The reduced affinities of HIV-1 protease for inhibitors seemed to be due to substantial decrease in the size of the active site and flap mobility. A correlation was found between the binding energy of inhibitors and their affinities for each mutant suggesting the distortion of the active site geometry in drug resistance by preventing effective fitting of inhibitors into the enzymes' active site. To overcome the problem of drug resistance of HIV-1 protease, designing inhibitors of variable functional groups and configurations is proposed.

## INTRODUCTION

The protease of human immunodeficiency virus type 1 (HIV-1 protease) is a homodimer aspartyl enzyme (E.C.3.4.23.16) made of two subunits each including 99 amino acids. The main function of the enzyme is to cleave HIV polyproteins, namely gag and pol, during viral replication in host cells. The protease active site consists of two triads of Asp25-Thr26-Gly27 (one from each subunit) with a C2 symmetry [1, 2]. The active site is covered by two flaps where residues 43-58 form two anti parallel beta strands connected by a turn. The flaps act gates for the enzyme active site to control entry/exit routes of substrates or inhibitors during enzyme activity [3, 4]. 

Inhibitors that mimic catalytic intermediates can bind to the enzyme in a competitive manner and prevent viral maturation and infection [5]. Therefore, HIV-1 protease has been a major target for anti- HIV drug designs. So far, nine such drugs have been approved for clinical AIDS treatment including, Saquinavir (SQV), Indinavir (IDV), Ritonavir (RTV), Nelfinavir (NFV), Amprenavir (APV), Lopinavir (LPV), Atazanavir (ATV), Tipranavir (TPV), and Darunavir (DRV) [6-9]. 

However, resistant HIV mutants have emerged which are capable of bypassing the inactivation by inhibitors. This is due to the genetic diversity and the fast mutating behavior of the HIV protease gene. The accelerated mutation of HIV-1 protease is supported by a high replication rate exceeding 108 virion/day as well as an error probability of 1/10,000 bases [10-12]. Many reports have shown that protease mutations have cumulative effects which confer reduced susceptibility to inhibitors [13, 14]. Measurements of binding energy have indicated that drug resistant mutants have lower affinities for inhibitors than for substrates [5, 15]. On other hand, decreased affinity for substrates does not cause a serious problem for polyprotein processing and viral maturation during viral attacks [10, 11, 16]. In vitro and in silico studies have indicated that drug-resistant mutations of HIV-protease occur in the hydrophobic core of the active site cavity, decreasing enzyme stability and specificity toward inhibitors [17-19]. These mutations commonly involve the substitution of more hydrophobic residues. Therefore, mutations which deter inhibitors of large P2 groups from fitting the protease active site, result in reduced inhibition [2, 20]. It has also been shown that in drug resistant mutants, protease Michaelis–Menten constant (Km) for substrates increases only 2-fold, while binding affinity of inhibitors decreases by an 85 to 2,000 factor [21, 22]. This represents a major obstacle that jeopardizes the success of HIV antiretroviral therapy using inhibitors. 

In the present work, we analyzed available data on mutant sequences from data banks to calculate the frequency of mutations and their distribution along the protease primary structure. We also aimed at determining hot spot protein residues that contained higher mutation incidences. In the next step, using molecular dynamic simulation, we studied the structural changes of mutants which confer resistance against inhibitors. We were hence able to shed light on protease resistance mechanisms against antiretroviral drugs in a bid to decipher the logic of drug resistance of the HIV virus at a molecular level. There is no doubt that decoding the drug resistance logic can help open up new horizons for drug design and protocol development for HIV therapy.

## MATERIALS AND METHODS

**Coordinate Structure Preparation: **A crystal structure of a wild-type protease with PDBID of 1MUI and no mutation was used as a wild-type structure throughout this study. Solved by X-Ray diffraction and refined at 2.0 Å resolutions, the wild-type structure was obtained from the Protein Data Bank, (www.rcsb.org/pdb) [23]. The same structure was used to prepare drug resistant mutants against inhibitors SQV, IDV, NFV, APV, LPV, ATV, TPV and DRV. The structures were constructed by introducing all reported drug resistance mutations using Swiss-Pdb Viewer software (http://www.expa sy.org/spdbv/) [24]. The structures were then named SRM, IRM, NRM, A^m^RM, LRM, A^t^RM, TRM and DRM respectively. Since insertion of one or more mutations in the aforementioned model structures produces only trivial conformational effects, mutants of maximum drug resistance were constructed using all known mutations. The models therefore, included all major and minor mutations as listed in Table 1. The models were assumed to help assess the ultimate effects of mutations.

**Table 1:** List of major (*) and minor mutations included in mutant models against FDA approved drugs. Mutations were prepared from Johnson et al., 2008 [24] and Weber & Agniswamy, 2009 reports [27]

**A** ^t^ **RM**	**LRM**	**TRM**	**IRM**	**SRM**	**DRM**	**NRM**	**A** ^m^ **RM**
L10I	L10I	L10V	L10I	L10I	V11I	L10I	L10I
G16E	K20M	I13V	K20M	L24I	V32I	D30N*	V32I
K20M	L24I	K20M	L24I	G48V*	L33F	M36I	M46I
L24I	V32I*	L33F*	V32I	I54V	I47V	M46I	I47V
V32I	L33F	E35G	M36I	I62V	I50V*	A71V	I50V*
L33F	M46I	M36I	M46I*	A71V	I54L*	V77I	I54V
E34Q	I47V*	K43T	I54V	G73S	T74P	V82A	G73S
M36I	I50V	M46L	A71V	V77I	I76V*	I84V	L76V
M46I	F53L	I47V*	G73S	V82A	I84V*	N88M	V82A
G48V	I54V	I54V	L76V	I84V	L89V	L90M*	184V*
I50L*	L63P	Q58E*	V77I	L90M*	L90M		
F53L	A71V	H69K	V82A*				
I54V	G73S	T74P*	I84V*				
D60E	L76V	V82L*	L90M				
I62V	V82A*	N83D					
I64L	I84V	I84V*					
A71V	L90M	L90M					
G73S							
V82A							
I84V*							
I85V							
N88S*							
L90M							
I93L							

**Simulation Settings: **Each constructed structure was placed in the center of a rectangular box with 5.30×4.82×7.06 nm dimensions. The boxes were then filled with SPCE water molecules using the genbox command of the GROMACS package so that each structure was covered with a water shell of 1.0 nm thickness. 

Molecular dynamic simulations were performed using the double-precision MPI version of GROMACS 4.5.5 installed on the UBUNTU version 12.04 with a 53A5 force field [25]. Net charges of simulated systems were analyzed by the preprocessor engine of the GROMACS package. System neutralization was done by adding equivalent numbers of negative chloride ions. Energy minimization was performed for hydrogen atoms, ions, and water molecules in a 1500-step energy minimization using the steepest descent method to minimize the system energy to at least 300kJ/mol. LINCS algorithm was used to apply constraint on bond lengths. The SETTLE algorithm was used to constrain the geometry of water molecules. The systems were then subjected to a short molecular dynamic with all-bonds restrained for a period of 500 ps before performing a full molecular dynamic without any restrains. Molecular dynamic simulations were carried out for 20ns at 37 ºC and 1 atmosphere of pressure. Berendsen, Thermostat and Barostat, were used for temperature and pressure coupling, respectively, together with the Particle Mesh Ewald (PME) method for electrostatic interactions. Time steps of 1 femtosecond were applied to all simulations. All simulations were done at neutral pH (Asp, Glu, Arg, and Lys ionized) [26]. 

In order to guarantee the reproducibility of simulations as recommended by GROMACS producers (http://www.gromacs.org/Documentation/Terminology/Reprodu cibility), we used the same binary input files on the same multi processor computer, the double precision version of MD integrator and the same MD parameters throughout the study. Moreover, the convergence of system energies to a finite value measured by the ratio of kinetic/total energy as well as the stability of the RMSD curve at the lag phase of simulation were considered to represent the attainment of an equilibrate state. To ensure the stability and reliability of our data, all simulation experiments were performed in triplicates. 

**Docking experiments: **Binding energy of inhibitors to the relevant mutants was calculated using Hex software version 5.1 (http://www.loria.fr/~ritchied/hex/) [28]. The physical fitness of inhibitors to their binding site before (as a wild-type structure) and after mutation (as an output structure of MD simulation) was also studied using dynamical correlation between native and complexed proteases. Docking results were scored based on their energy, and the first 100 solutions were averaged and used as binding energy of inhibitors to the protease.

**Statistical Analysis: **The resulting data were analyzed statistically using the Statistical Package for the Social Sciences (SPSS, version 15, Inc., Chicago, IL). The differences between parameters were considered significant at P<0.05.

## RESULTS AND DISCUSSION

**Figure 1:**
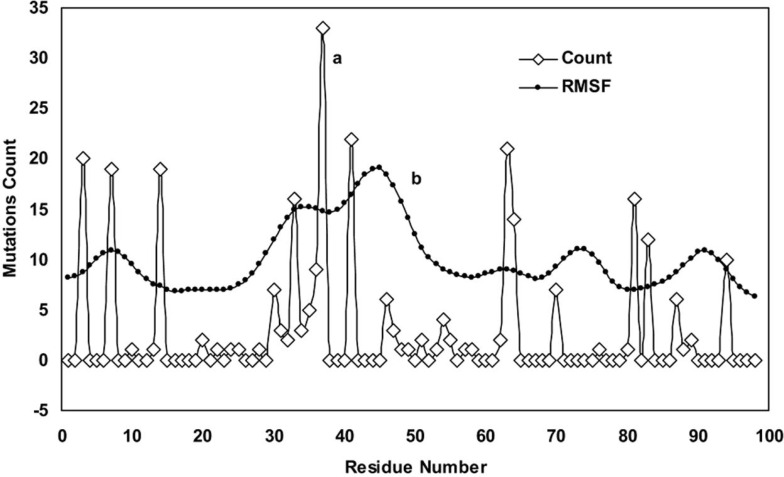
Curve **a**: counts of mutations found along protein chains that calculated from all available sequences in PDB data bank (http://www.rcsb.org/pdb). Curve **b: **RMSF curve for wild-type protease obtained from 20ns simulation at 37°C, 1 atmosphere of pressure, pH7 and in SPCE water box

Curve **a** in Figure 1 shows the mutation counts found along the protein chains (from residue 1 in the N-terminal end to residue 99 at the C-terminal end) in 91 protease mutant structures obtained from the Protein Data Bank (http://www.rcsb.org/pdb). As indicated, the mutations are not uniformly distributed through the protein chains; instead, they are concentrated in certain positions e.g. around residues 8, 40 and 70-90 as described previously [29]. The distribution pattern shows some critical points with high mutations incidences. Curve **b** in Figure 1 is the superposed Root Mean Square Fluctuation (RMSF) curve of the wild-type protease obtained by MD simulation experiments. Peaks on the RMSF profile belong to more fluctuating or more flexible points in the protein structure. By comparing curves **a** and **b**, we found that mutations occurred more predominantly in regions that corresponded to flexible points on the protease with a higher RMSF. Therefore, occurring in the vicinity of the protease hot points, mutations seem to have primarily affected the flexibility and specificity of the enzyme toward inhibitors.

Using the *hydrophobicity* scale of Kyte-Doolittle [30], we also calculated the total change in protease hydrophobicity upon inserting the drug resistance mutations listed in Table 1 and summarized them in Table 2. As depicted, all mutants showed increased hydrophobicity when contrasted to the wild-type protease. 

**Figure 2:**
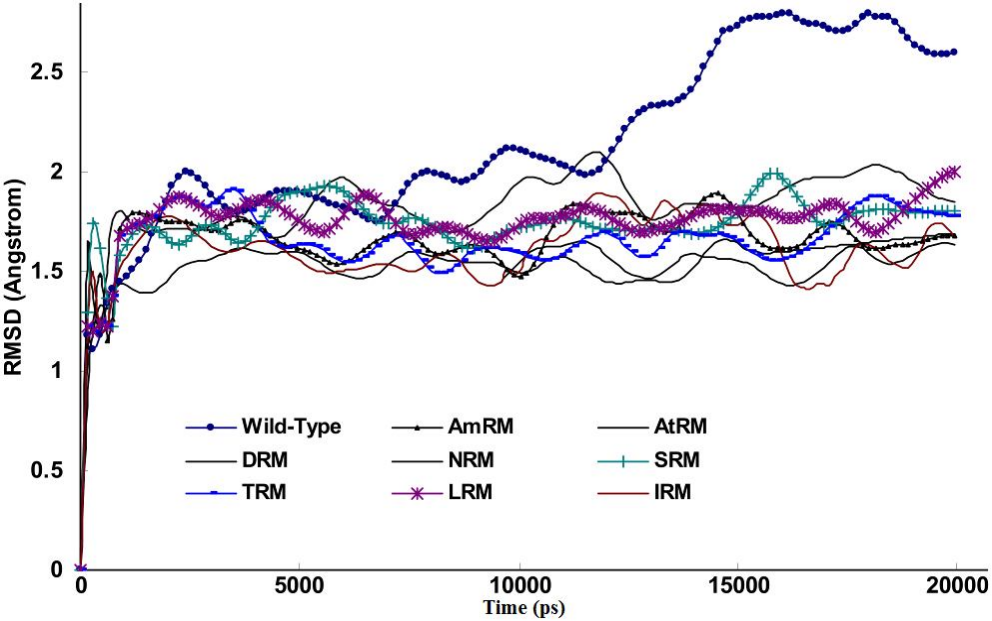
Curve of RMSD for wild-type and drug resistant mutants extracted from simulations experiments for 20ns period at 37°C, 1 atmosphere of pressure, pH7 and in SPCE water box

Figure 2 shows the Root Mean Square Displacement (RMSD) curve of the wild-type protease and drug resistant mutants during simulations. Using the coordinate file of the wild-type (1MUI) as control as a starting structure for the construction of drug resistant mutants, we were able to study gradual alterations of the protease structure induced by mutations on a time step basis. This could not have been as informative if mutants of different structures and coordinates were obtained from the Protein Data Bank for the same purpose. Figure 2 indicates that for mutants, the progression of the RMSD curve was significantly limited compared to the wild-type protease. This means that mutations retained their structural changes while the protein sensed the surrounding conditions of 37°C, a neutral pH, and a 1 atmosphere pressure throughout the MD simulations. The difference between the final RMSDs for the wild-type and other mutations is as small as 0.9Å. This implies that mutations do not experience vast structural alterations, thus, as mentioned previously, the resulting structures are useful for comparative studies [31-32].

**Table 2:** Total changes in Kyte-Doolittle index caused by mutations listed in Table Ia with positive values reveal increase in hydrophobicity

	**Kyte-Doolittle Index**
A^t^RM	+23.59
LRM	+3.11
TRM	+71.71
IRM	+27.56
SRM	+9.04
DRM	+11.30
NRM	+49.21
A^m^RM	+0.97

**Figure 3:**
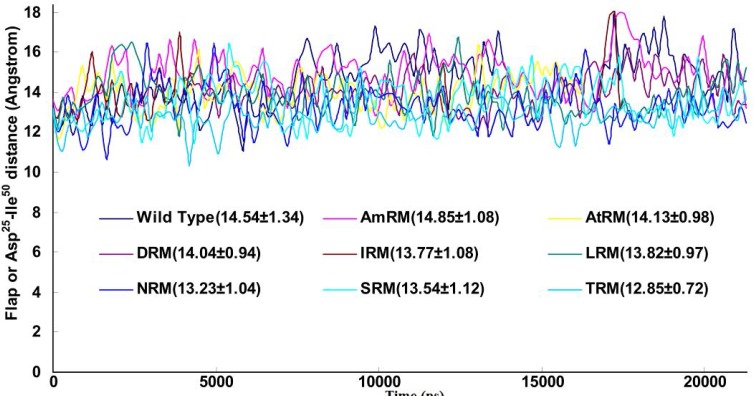
Plots of distances between beta carbons of Asp^25^ from one chain to alpha carbon of Ile^50^ of the same chain during simulation for 20ns simulation at 37°C, 1 atmosphere of pressure, pH7 and in SPCE water box

Bearing this finding in mind, we examined further parameters in order to survey sub-global alterations exerted by mutations to obtain mechanistic information. One such parameter is the distance between the beta carbon of Asp^25^ and the alpha carbon of Ile^50^ on the same chain (Asp^25^-Ile^50^ distance), which has been widely used as an index for flap states. Using the g_mindist command, the Asp^25^-Ile^50^ distance (as a curve plus average ± SD) was calculated and plotted against simulation time in Figure 3. As indicated, there was no obvious difference among the wild-type and the mutants in terms of their flap states (Asp^25^-Ile^50^ distance). This means that mutations did not affect either flap opening or closing. Hence, we postulate that drug resistance is unlikely to be mediated by flap alteration.

**Figure 4:**
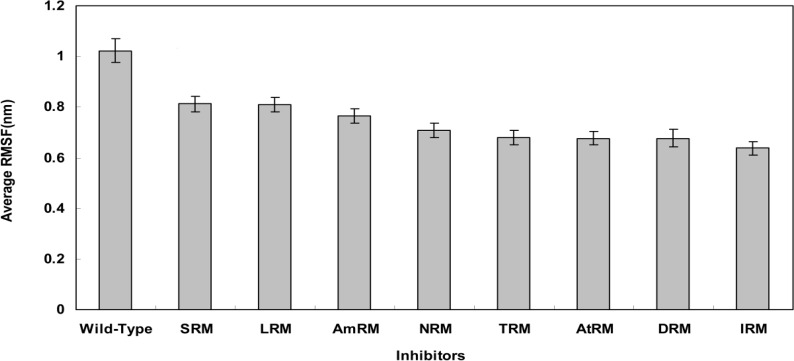
Plot of RMSF (Average±SE) of protease flaps (residues 42-52) for wild-type and resistant mutants protease extracted from simulation trajectory for 20ns period at 37°C, 1 atmosphere of pressure, pH7 and in SPCE water box

However, as previously indicated, the calculation of RMSF (average ± SD) values of residues 42-52 provides a better measure for flap flexibility [33]. Figure 4 depicts the average RMSF for residues 42-52 during simulation period. Flap flexibility significantly decreased in mutants compared with the wild-type (P<0.05). Since flap flexibility is essential for accessing the active site, all mutants with reduced flexibility are expected to exhibit decreased sensitivity to inhibitors [34].

**Figure 5:**
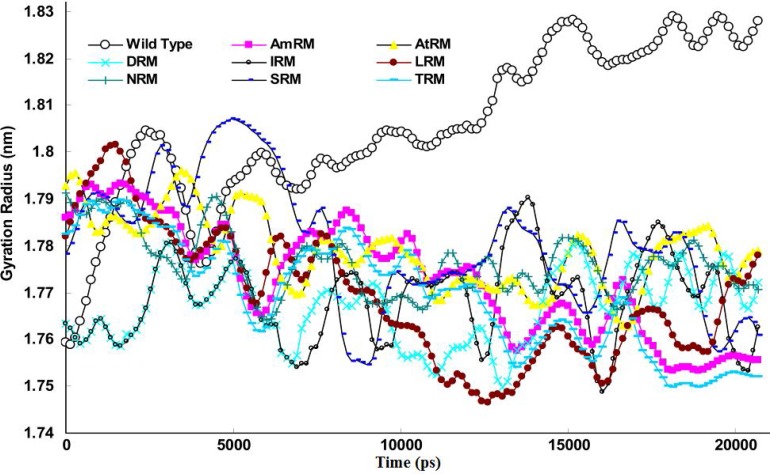
Plot of gyration radius for wild-type and drug resistant mutants with time obtained from 20ns simulation at 37°C, 1 atmosphere of pressure, pH7 and in SPCE water box. The curve is calculated as average from triple experiments

The gyration radius of the protein and its changes during simulation are other useful parameters for protein structure analysis. These parameters were extracted from a trajectory file using the g_gyrate command of the GROMACS package. Figure 5 depicts the averaged curve of triple experiments for the gyration radius of the wild-type and mutant proteases over time. The figure indicates an apparent decrease of gyration radius for mutants but not for the wild-type protease. Based on this curve, we may postulate that mutations increase the protein folding but decrease the size of both the protein and its cavities (e.g. the active site).

**Figure 6:**
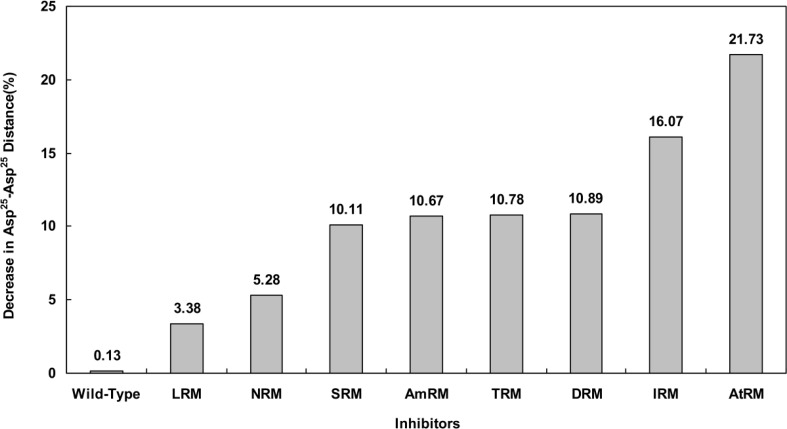
Percent of decrease in the distance between alpha carbon of Asp^25^ from chain A and alpha carbon of Asp^25^ from chain B obtained from simulation for 20ns period at 37°C, 1 atmosphere of pressure, pH7 and in SPCE water box.

As a diagonal parameter reflecting the size of the active site, we calculated the distance between one Asp^25^ residue of the enzyme active site triad from chain A and the same residue from chain B (Asp^25^A-Asp^25^B) during the simulation using the g_mindist command. We then implemented the measurement of this distance as an indication for active site compression or expansion in the presence or absence of mutations. Figure 6 presents the variation of Asp^25^A-Asp^25^B distance amongst different mutants. As depicted, Asp^25^A-Asp^25^B distances tend to decrease for all studied mutants. Figure 6 shows that, upon simulation, the mutants experienced about 3 to 21 percent decrease in their Asp^25^A-Asp^25^B distances compared to only 0.13% decrease for the wild type. In this context, mutants of the leucin-rich motif (LRM) and arginine-rich motif (A^t^RM) showed minimum and maximum compression in their active site cavity, respectively. It is important to mention that this finding does not necessarily mean that the LRM mutant exhibits lower drug resistance than the A^t^RM mutant when administered clinically. This is because the size of the protease active site is not the only determinant of resistance against HIV drug therapy. 

Nevertheless, the shrinkage of the active site was expected to affect the protease-inhibitors' binding energy. To find out about the change in binding energy, inhibitors were docked to their relevant mutants using HEX software. The calculated binding energies of inhibitors to the wild-type and mutants are listed in Table 3 (as means ± SD). The binding energies of all inhibitors to the mutants rather than the wild-type protease have shown decreases (reported in the table as percentages). 

**Table 3:** Binding energies of inhibitors to wild-type and to their correspondent mutants presented as average ± SD

	Wild Type (KJ/Mol)	Mutants (KJ/Mol)	Decrease (%)
A^m^RM	-263.47±11	-234.5±9	11
A^t^RM	-273.41±8.6	-260±10	5
DRM	-264.19±6.5	-251.33±6	5
IRM	-295.52±14	-262.68±10	11.11
LRM	-364.73±14	-244.26±12	33
NRM	-355.96±9.2	-257.01±10	27.7
SRM	-228.26±12	-203±9	11
TRM	-348.51±12	-317.45±8	8.9

**Figure 7:**
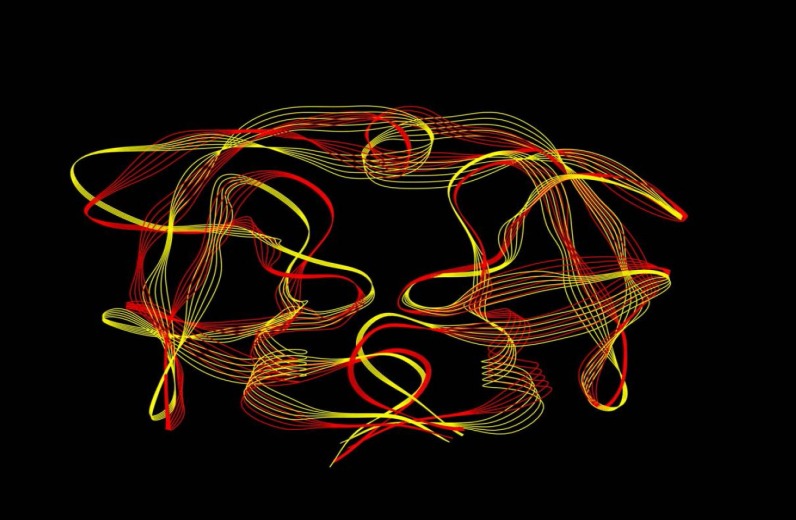
Schematic representation of HIV-1 protease tertiary structures for wild-type (yellow) and mutant of TRM (red) extracted from the last frame of trajectories for 20ns simulations at 37°C, pH7, 1 atmosphere of pressure and in explicit water box

In order to illustrate the conformational deviation of the model structures, we extracted the last frames of the wild-type protease and the mutants from MD trajectories. We then superposed the tertiary structure of one protease mutant, namely the TRM (red model), on the wild-type (yellow model) structure (Fig. 7) to construct a representative model. As depicted, there are no differences between the wild-type and the mutants in terms of flap status as already shown in Figure 2.

The emergence of drug resistant mutants remain the main obstacle in HIV therapy using inhibitors such as SQV, IDV, RTV, NFV, APV, LPV, ATV, TPV and DRV. The drug resistance arises as a consequence of the rapid mutation of the HIV genome and its response to selection pressure induced by the presence of inhibitors in the replication medium. Mutations of HIV-1 protease may be classified into two types: 

1- Primary or major mutation, which takes place primarily in the active site residues. This kind of mutation affects the interaction of inhibitors with active site residues. An example is the mutation of D30N that affects the interaction between P_2_/P_2_ groups of inhibitors and relevant groups on the enzyme active site. Other examples of primary mutations are the mutation of K45I that affects the interaction between P_3_/P_3_ and P_4_/P_4_ groups and the mutation of V82S that affects P_1_/P_1_ groups of inhibitors and their counter groups on the protease active site [13]. A single primary mutation normally results in resistance against a specific drug. An example is the D30N mutation, which causes resistance to the NFV drug. Many studies have reported single mutations that simultaneously decreased protease affinity and sensitivity to drugs [31-32]. 

2- Secondary or minor mutation, which occurs in the distal places of the enzyme active site. This kind of mutation causes a lower degree of drug insensitivity by the protease. However, it may play a supporting role for primary mutations by decreasing enzyme susceptibility to inhibitors [11, 34]. 

Given the mechanism of drug resistance, protease mutations may also be classified into three classes: 

i- Active site mutations that directly affect drug-active site interaction (see below). 

ii- Mutations at the dimer interface that affect dimer stability against dissociation. Mutation of L24I which causes resistance against SQV, IDV, NFV, LPV and ATV is an example of such mutations. Other examples are the mutation of I50V against APV and DRV and the mutation of F53L against LPV and ATV. 

iii- Distal mutations are those reducing flap movement and flexibility which confers lower affinity for inhibitors [11, 35-36]. Examples are the mutation of I54M against DRV, the mutation of L90M against NFV and the mutation of V88D against NFV, ATV. This kind of mutation is the most frequently seen within protease mutants [27]. 

Considering our results as well as those of other studies, it is obvious that the mechanism of drug resistance is mutant-dependent, showing complete variability at the molecular level. Therefore, more precise studies are still needed to help understand the exact mechanisms of resistance. Unless the logic behind variable patterns of mutations is clarified, newly synthesized drugs remain exposed to resistance risk. If we are to draw a new hypothesis useful in solving the puzzle of protease resistance, the following arguments sound of prime importance: 

1- Flaps adapt to at least one of the following three possible conformations: closed, for liganded state (enzyme bind to substrate or inhibitors) with an Asp^25^-Ile^50^ distance lower than 10Å, semi-opened and opened with an Asp^25^-Ile^50^ distance more than 15 Å [31, 33, 37, 38]. 

2- Drug resistant mutants have decreased flexibility in flap regions and exhibit a decreased rate of flap closure or an increased rate of flap opening [32, 34, 35]. 

3- Mutations frequently decrease the binding affinity of the enzyme to its inhibitors. 

In light of the above arguments, we may now discuss our results in order to build a hypothesis on the logic behind the mutation pattern that leads to drug resistance. Figure 1 (curve **a) **shows that protease mutations emanated from natural polymorphism or induced by drugs are not randomly distributed along the protein sequence. On the other hand, figure 1 (curve **b)** depicts the RMSF curve of the wild-type protease along its sequence. Examining both curves **a** and **b,** we found that mutations were likely inserted in flexible points of the protease which have higher RMSF values. These kinds of viral mutations produce more conformational changes in the protease and hence provide the virus with structures of greater conformational and functional diversities to choose from in order to adapt to new conditions. Our previous works showed that the fluctuation of RMSD curves of the wild-type protease or proteases with single or dual mutations were somehow similar suggesting that the increased number of mutations did not exert major structural changes [32]. However, the plotted RMSD curves in figure 2 indicate remarkable differences amongst wild-type and drug resistant mutants: those with lower propagating RMSD manifested stronger resistance against structural alterations induced by the MD force field. Lower RMSD may be attributed to the more stable and probably more compacted structures of the protease mutants. The data in figure 3 suggests semi open conformations for both wild-type and mutants for which the enzyme experiences a conformational free state with lower binding affinities for drugs. Calculating flap flexibility by determining the average RMSF for residues 42-52 (Fig. 4) indicated that flap flexibility of mutants decreased up to 20-30% of that of wild types. As reported earlier, this effect attenuates enzyme affinity and even specificity for their corresponding inhibitors [35, 36, 39]. Determination of gyration radius (Fig. 5) for the wild types and mutants indicated that mutations caused a slight decrease in the radius of mutants or blocked the increase in gyration radius of mutants over time. The curve in figure 5 also provides further evidence that mutants have more compacted and stable structures. Figure 6 depicts 3% to 21% reduction in distance between the Asp^25^A and Asp^25^B of different mutants as a measurement of their binding site. This finding reconfirms the more compacted structures of mutants. In fact, both figures 5 and 6 provide evidence that mutants have more compacted structures and shrunk active sites. Furthermore, we measured the binding energies of mutants to their corresponding inhibitors in order to verify whether mutants were of lower affinities. The data presented in Table 2 shows a 5 to 27% decrease in binding energies (binding affinities) of mutants to their inhibitors.

Taking into account the results and those of other studies, we may suggest a mechanism for drug resistance as follows. Resistance mutations involve the substitution of hydrophobic amino acids primarily in flexible points of the protease chains (Table 1 and Fig. 1) resulting in a protease of a more compacted structure and decreased flap flexibility. However, concomitant decrease in the size of the active site and flap flexibility may also be involved in the reduction of the binding affinity of the protease for inhibitors, hence resulting in drug resistance. Therefore, we find it logical to propose that inhibitors of different configurations, sizes and functional groups may be used if we are to overcome drug resistance.

## Notes

**Acknowledgements**: The authors gratefully acknowledge the financial support and services provided by Shahid Chamran University and Tarbiat Modares University.
